# Effect of sleep quality on weaning from mechanical ventilation: A scoping review

**DOI:** 10.2478/jccm-2024-0043

**Published:** 2025-01-31

**Authors:** Hana Locihová, Darja Jarošová, Karolína Šrámková, Jana Slonková, Renáta Zoubková, Klára Maternová, Karel Šonka

**Affiliations:** Department of Anesthesiology, Resuscitation and Intensive Care Medicine, Faculty of Medicine, University of Ostrava, Ostrava -Vítkovice, Czech Republic; Department of Nursing and Midwifery, Faculty of Medicine, University of Ostrava, Ostrava - Vítkovice, Czech Republic; Department of Nursing and Midwifery, Faculty of Medicine, University of Ostrava, Ostrava - Vítkovice, Czech Republic; Department of Neurology, University Hospital Ostrava, Ostrava - Vítkovice, Czech Republic; Department of Neurology, University Hospital Ostrava; Department of Clinical Neurosciences, Faculty of Medicine, University of Ostrava, Ostrava - Vítkovice, Czech Republic; Department of Anesthesiology, Resuscitation and Intensive Care Medicine, Faculty of Medicine, University of Ostrava; Department of Anesthesiology, Resuscitation and Intensive Care Medicine, University Hospital Ostrava, Ostrava - Vítkovice, Czech Republic; 2^nd^ Department of Surgery – Department of Cardiovascular Surgery, First Faculty of Medicine, Charles University, General University Hospital in Prague, Prague, Czech Republic; Department of Neurology and Center of Clinical Neurosciences, First Faculty of Medicine, Charles University, General University Hospital in Prague, Prague, Czech Republic

**Keywords:** extubation, mechanical ventilation, scoping review, sleep-weaning

## Abstract

**Introduction:**

Mechanically ventilated patients have disturbed sleep.

**Aim of the study:**

To explore whether there is a relationship between successful or unsuccessful weaning of patients and their sleep quality and circadian rhythm.

**Materials and Methods:**

A scoping review. The search process involved four online databases: CINAHL, MEDLINE, ProQuest, and ScienceDirect. Original studies published between January 2020 and October 2022 were included in the review.

**Results:**

Six studies met the inclusion criteria. These studies showed that patients with difficult weaning were more likely to have atypical sleep, shorter REM sleep, and reduced melatonin metabolite excretion. Muscle weakness was an independent factor associated with prolonged weaning from mechanical ventilation and was significantly more frequent in patients with atypical sleep. Heterogeneous patient samples and the methodology of the studies hamper a clear interpretation of the results.

**Conclusions:**

A relationship was found between abnormal sleep patterns, reduced melatonin metabolite (6-sulfa-toxymelatonin) excretion, and unsuccessful weaning. However, the causality is not clear from the existing research.

## Introduction

Weaning refers to liberating the patient from mechanical ventilation. It is a rather individual process, the success of which depends on many factors such as the patient's condition and comprehensive assessment, adequate timing, adaptation of the patient's breathing pattern, and many others. The 2005 International Consensus Conference in Intensive Care Medicine proposed a classification of patients into three groups (simple weaning, difficult weaning, and prolonged weaning) according to the difficulty and duration of the weaning process. The classification considers the number of spontaneous breathing trials, timing, and results [1]. Approximately 70% of patients experience simple weaning, while the others fall into the difficult and prolonged groups [2]. Repeated weaning attempts can account for a significant proportion of the total time on mechanical ventilation and prolong the patient's stay in the intensive care unit (ICU) [3].

In current medical practice, there is a trend toward standardized procedures that lead to earlier weaning and include four main points: (a) daily assessment of readiness for extubation with a spontaneous breathing trial, (b) early mobilization, (c) rehabilitation, and (d) periodic sedation interruptions [4].

Sedation is one of the main determinants of patient-ventilator synchronization. Sedatives and analgesics are indispensable in treating critically ill patients, but the commonly used drugs modify physiological functions, including sleep and wakefulness. Although they induce a behavioral state similar to sleep, it is not natural sleep. Benzodiazepines prolong the N2 sleep stage (N2) and shorten the N3 sleep stage (N3) (the latter formerly referred to as NREM3 and NREM4) and REM sleep [5]. Propofol shortens the N3 and REM sleep [6]. Data on the effects of opioids on sleep are inconsistent, probably due to different doses and types of opioids. The prevailing view is that opioids shorten N3 and REM sleep [7].

Another reported effect of benzodiazepine and opioid therapy is the development of central sleep apnea due to its depressor effects on the central nervous system [8,9]. Moreover, opioid administration during mechanical ventilation increases upper airway collapsibility during wakefulness and sleep [10]. There is a relationship between the severity of obstructive sleep apnea (OSA) assessed by the apnea-hypopnea index shortly after extubation and the dose of opioids that ICU patients receive 24 hours before extubation [11]. The effect of medications administered during ICU stay on sleep breathing cannot be accurately separated from premorbid OSA, which is very common (9–38%) in the general population [12], increases with age [13], and remains undiagnosed in 80% of patients undergoing surgery [14]. In addition, OSA doubles the risk of postoperative complications [12].

As many as 40% of ICU patients of all ages require mechanical ventilation, with the proportion being highest in those aged 65 years and older [15]. The advancing age may increase the impact of medications and comorbidities like OSA on sleep quality. Aging is associated with sleep duration and architecture changes, more awakenings, lower regenerative capacity, and difficulty adapting to a new environment [16]. A relationship has been found between disturbed sleep and muscle weakness in critically ill patients [17].

The effect of sleep shortening or poor sleep quality on respiratory muscle function has not been sufficiently studied. The only two experimental studies with healthy volunteers exposed to sleep deprivation found an association with impaired respiratory muscle performance, reduced respiratory effort, and diaphragmatic dysfunction [18,19]. These studies support the notion that sleep deprivation contributes to neuro-muscular weakness that may manifest during weaning from mechanical ventilation.

Even though there is a consensus on the importance of sleep in general and in hospitalized patients, little attention has been paid in the literature to a comprehensive examination of its influence on weaning (or extubation). Several parameters characterize sleep quality, and there are several reasons for weaning failure (mode of ventilation, underlying disease, medication, etc). This review aimed not to find a single clinically relevant sleep parameter important for weaning (given its complexity, we do not even consider this possible) but to identify the most important sleep and circadian rhythm abnormalities that may impair weaning.

## Materials and methods

### Design

A scoping review was undertaken according to the five-stage framework [20]: (1) identifying the research question; (2) identifying relevant studies; (3) study selection; (4) charting the data; and (5) collating, summarizing, and reporting the results. Reporting of the scoping review was guided by the Preferred Reporting Items for Systematic Reviews and Meta-Analyses (PRISMA) Extension for Scoping Reviews checklist [21].

### Research Question

Is there a relationship between sleep quality, circadian rhythm, and weaning success from mechanical ventilation?

### Inclusion and exclusion criteria

Publications meeting the following criteria were included in the scoping review: (a) date of publication between January 2000 and October 2022; (b) original study; (c) sleep quality as a factor influencing weaning from mechanical ventilation or extubation in hospitalized patients as the main topic; (d) objective and/or subjective sleep quality assessment; and (e) English language. The exclusion criteria were as follows: (a) patients younger than 18 years; (b) patients with neuromuscular diseases; (c) studies focusing only on the effect of mechanical ventilation modes on sleep quality; (d) home mechanical ventilation; and (e) reviews.

### Resources and search strategies

We searched four literature databases: Cumulative Index to Nursing and Allied Health Literature (CINAHL), MEDLINE, ProQuest, and ScienceDirect.

The following search terms were used: *patients*, *in-patients*, *hospitalized*, *quality of sleep*, *sleep disturbance*, *weaning*, *spontaneous breathing trial*, and *extubation*. The search terms were combined using the Boolean operators AND and OR. The same search criteria were applied to each database. We used the results of available relevant original studies to verify the objectives and provided further analysis.

### Study selection and data extraction

A total of 252 studies were identified in the selected databases by three researchers and one librarian. Studies were reviewed independently by two researchers. Sixty-seven studies were removed as duplicates. Based on titles and abstracts, the initial screening validated 185 studies, of which 151 were excluded for lack of relevance to the topic of concern. Another 28 studies were excluded during full-text assessment as they did not meet the inclusion criteria. We checked the reference lists of the remaining six papers for possible missing studies. Studies were selected and classified according to the PRISMA flow diagram (Figure 1). We present primary data extraction from the selected six papers, including information on authors, country of origin, research design, study objectives, data processing forms, research findings, and conclusions in Tables 1 and 2. Next, we provided an analysis of the variables of interest and a synthesis of the findings.

**Table 1:** Study characteristics – effect of hospitalized patient’ sleep quality (objectively measured) on weaning from mechanical ventilation

**Author(s), year, country**	**Objective(s)**	**Study design/patients Definitions of groups**	**Methods (parameters assessed)**	**Use of an alternative classification for sleep assessment**	**Use of sedation during patient monitoring**	**Results**	**Sleep and weaning outcome**	**Other weaning predictors**	**Conclusion (sleep concerning weaning)**
Huttmann et al., 2017, Germany	To assess sleep quality in tracheotomized patients undergoing prolonged weaning	A cross-sectional study of 19 patients undergoing prolonged weaning at a specialized weaning unit of a pneumology department Groups: *“successful weaning”* and *“unsuccessful weaning”*	*Objective measurements:* PSG (10 pm – 6 am) Gas exchange monitoring *Subjective evaluation:* Sleep quality and SRI *Other parameters assessed:* Days on invasive MV	**NO**	**NO**	7 patients (36.8%) *successful weaning* vs. 12 (63.2%) *unsuccessful weaning* Days on MV: 39 (SD 22) *successful weaning* vs. 187 (SD 335) *unsuccessful weaning*; p = 0.473	No significant difference in sleep quality between the *successful weaning* and *unsuccessful weaning*groups in PSG A decreased amount of REM sleep: 9.1 (SD 6.3) vs. 5 (SD 8.4), respectively	No significant difference in nocturnal gas exchange between the groups	There was no difference between *successful* and *unsuccessful weaning* groups of patients undergoing prolonged weaning.

Dres et al., 2019, Canada	To determine whether abnormal sleep or wakefulness is associated with SBT outcome	A prospective multicenter study of 44 (enrolled; 37 with adequate signals) intubated mechanically ventilated patients with an SBT planned for the next day at 3 ICUs Groups: *“failed SBT,” “successful SBT (extubation),”* and *“successful without extubation”*	*Objective measurements:* PSG (5 pm – 8 am) EEG markers (ORP index + hemispheric correlation [ICC R/L ORP]) *Subjective evaluation:* Delirium (CAM-ICU) *Other parameters assessed:* SOFA score, days on MV, length of ICU stay	**YES**	**YES**	11 patients (30%) *successful SBT (extubation)* vs. 8 (21%) *successful without extubation* vs. 18 (49%) *failed SBT* Days on MV: 10.4 (SD 8.6) *successful SBT (extubation)* vs. 5.0 (SD 2.5) *successful without extubation* vs. 4.4 (SD 3.2) *failed SBT*; p < 0.01	No significant difference in sleep architecture between the groups shown by PSG Abnormal sleep patterns are present but not significant More time with ORP > 2.0 and > 2.2 in the *successful SBT (extubation)* group than in the other two; p < 0.01. Differences in R/L ORP ICC: 0.80 (SD 0.16) *successful SBT (extubation)* vs. 0.80 (SD 0.15) *successful without extubation* vs. 0.54 (SD 0.26); p = 0.006	SOFA score: 7 (SD 3) *successful SBT (extubation)* vs. 8 (SD 3) *successful without extubation* vs. 6 (SD 3) *failed SBT*; p = 0.32 Delirium: 3 (27%) *successful SBT (extubation)* vs. 2 (25%) *successful without extubation* vs. 0 (0%)*failed SBT*; p = 0.06	Although abnormal sleep patterns were noted, there was no association between sleep architecture changes and weaning. However, a detailed analysis of derived EEG markers (ORP, R/L ORP ICC) identified these parameters helpful in predicting SBT success.

Thille et al., 2018, France	To assess the impact of sleep alterations on weaning duration	A prospective single-center study of 45 intubated patients with at least one SBT failure at a medical ICU Groups: *“short weaning”* (< 3 days) and*“prolonged weaning”* (> 3 days)	*Objective measurements:* PSG (1–4 nights) EEG reactivity at eyes opening during wakefulness assessed by a neurologist *Subjective evaluation:* Delirium (ICDSC) ICU-AW (MRC score < 48) *Other parameters assessed:* SOFA score, days on MV, length of ICU stay, mortality in ICU	**YES**	**YES**	27 patients (60%) *short weaning* vs. 18 (40%) *prolonged weaning* Days on MV (median, IQR): 8 (4–13) *short weaning* vs. 13 (15–20) *prolonged weaning*; p = 0.19	Weaning duration is significantly longer in patients with atypical sleep compared with those with normal sleep (median, IQR): 5 (2–8) vs. 2 (1–2); p < 0.001 and independently associated with prolonged weaning: OR = 13.9, 95% CI 3.2–85.7; p = 0.001 Weaning duration is significantly longer in patients with no REM sleep compared with the others (median, IQR): 4 (2–7) vs. 2 (1–2); p = 0.03	Delirium: 10 (37%) *short weaning* vs. 6 (33%)*prolonged weaning*; p > 0.99 SOFA score (median, IQR): 3 (2–3) *short weaning* vs. 4 (3–6) *prolonged weaning*; p = 0.02 ICU-AW: 9 (33%) *short weaning* vs. 12 (71%) *prolonged weaning*; p = 0.03	Patients with atypical sleep or no REM sleep had markedly longer weaning duration than those with normal sleep. Atypical sleep was associated with prolonged weaning (a strong predictor).

Thille et al., 2021, France	To assess whether sleep alterations after extubation are associated with an increased risk of reintubation	A prospective observational single-center study of 52 extubated patients at a medical ICU Groups: *“extubation success”* and *“reintubation”*	*Objective measurements:* PSG (afternoon to next morning) *Subjective evaluation:* ICU-AW (MRC score < 48) Delirium (ICDSC) *Other parameters assessed:* Mortality, SOFA	**YES**	**YES**	44 patients (85%) *extubation success* vs. 8 (15%) *reintubation* Days on MV (median, IQR): 3 (2–7) *extubation success* vs. 9 (5–15) *reintubation*; p = 0.043	Reintubation rates 21% (7/33) in patients with no REM sleep and 5% (1/19) in patients with REM sleep, difference −16% (95% CI −33% to 6%); p=0.23 No statistically significant changes in the other PSG sleep parameters between the groups	SOFA score (median, IQR): 3 (2–4) *extubation success* vs. 3 (2–5) *reintubation*; p = 0.919 Delirium: 4 (10%) *extubation success* vs. 4 (33%) *reintubation*; p = 0.08 ICU-AW: 11/36 (30%) *extubation success* vs. 6/8 (86%) *reintubation*; p = 0.009	Absence of REM sleep influenced the risk of reintubation in the ICU.

Dessap et al., 2015, France	To assess the impact of delirium during weaning and associated alterations in the circadian rhythm	An observational multicenter study of 70 patients intubated for over 24 hours in an ICU Groups: *“successful extubation with delirium”* and *“successful extubation without delirium”*	*Objective measurements:* Excretion of the melatonin urinary metabolite 6-SMT during weaning *Subjective evaluation:* Delirium (CAM-ICU) *Other parameters assessed:* SOFA score, days on MV, mortality in ICU	**NO**	**YES**	43 patients (61.4%) *successful extubation with delirium* vs. 24 (34.3%) *successful extubation without delirium*; 3 comatose patients (4.3%) Days on MV (median, IQR): 4.1 (2.6–7.4) *successful extubation with delirium* vs. 2.8 (1.6–6.9) *successful extubation without delirium*; p = 0.133	Reduced excretion of 6-SMT (ng) in patients with delirium (median, IQR): 20.212 (23.207–39.920) vs. 18.880 (11.462–27.325); Interaction between delirium and 6-SMT secretion: F statistic = 2.65; p = 0.019	SOFA score (median, IQR): 8.0 (6.0–11.0) *successful extubation with delirium* vs. 5.5 (4.0–7.8) *successful extubation without delirium*; p = 0.1 More complications during weaning in patients with delirium: 40 (93%) vs. 15 (63%); p = 0.02 (OR 5.95, 95% CI 1.26–28.13; p = 0.021) Successful extubation is less likely in patients with delirium: HR 0.54, 95% CI 0.30–0.95; p = 0.02 Alcohol abuse (median, IQR): 11 (25.6%) *successful extubation with delirium* vs. 1 (4.2%) *successful extubation without delirium*; p = 0.044	*Urinary 6-SMT was associated with alterations in the circadian rhythm in patients with delirium and was identified as a measurable marker of the circadian rhythm.*

CAM-ICU: Confusion Assessment Method for the Intensive Care Unit, CI: confidence interval, EEG: electroencephalogram, HR: hazard ratio, ICC: intraclass correlation coefficient, ICDSC: Intensive Care Delirium Screening Checklist, ICU: intensive care unit, ICU-AW: intensive care unit-acquired weakness, IQR: interquartile range, MRC: Medical Research Council, MV: mechanical ventilation, ng: nanogram, OR: odds ratio, ORP: odds ratio product, PSG: polysomnography, REM: rapid eye movement, R/L: right/left, SBT: spontaneous breathing trial, SD: standard deviation, SOFA: Sequential Organ Failure Assessment, SRI: Severe Respiratory Insufficiency, 6-SMT: 6-sulfatoxymelatonin

**Table 2.** Study characteristics – effect of hospitalized patient' sleep quality (subjectively evaluated) on weaning from mechanical ventilation

**Author(s), year, country**	**Objective(s)**	**Study design/patients Definitions of groups**	**Methods (parameters assessed)**	**Use of sedation during patient monitoring**	**Results**	**Sleep and weaning outcome**	**Other weaning predictors**	**Conclusion (sleep concerning weaning)**
Chen et al., 2015, Taiwan	To investigate the predictors of sleep quality and successful weaning	A cross-sectional study of 94 patients in the process of weaning from MV at 3 respiratory care centers Groups: *“weaned group”* (successfully weaned within 72 hours) and *“nonweaned group*“	*Subjective evaluation:* The first two parts of a questionnaire: demographic (age, gender) and clinical (co-existing chronic illnesses, alcohol drinking, use of hypnotics, tracheotomy, albumin, days on MV) information Third part: disease severity (APACHE II and GCS scores) Fourth part: sleep (VSH score) *Other parameters assessed:* Days on MV	YES	53 patients (56.4%) *weaned group* vs. 41 (43.6%) *non-weaned group* Days on MV: 37.9 (SD 17.8) *weaned group* vs. 42.5 (SD 20.4) *non-weaned group*; p = 0.240	Sleep quality was better in the *weaned group* than in the *non-weaned group*: 45.9 (SD 15.3) vs. 36.1 (SD 16.5); p = 0.004 Sleep quality negatively influenced by disease severity (APACHE II score: b = −1.323, 95% CI −2.052 to −0.593; p < 0.001), use of hypnotics (b = −10.707, 95% CI −16.718 to −4.696; p < 0.001), and 3–4 co-existing illnesses (b = −9.905, 95% CI −17.734 to −2.077; p < 0.14	The *weaned group* characterized by younger patients (p = 0.038) with higher GCS scores (p = 0.05) and less severe disease (p < 0.001) Other factors identified as independent predictors of weaning (based on regression analysis): APACHE II score (OR = 1.644, 95% CI 1.150–2.351; p < 0.06), GCS score (OR = 0.810, 95% CI 0.695–0.944; p < 0.07), and alcohol use (OR = 0.208, 95% CI 0.063–0.689; p < 0.09)	Sleep quality was identified as a significant predictor of successful weaning from MV.

Huttmann^*^ et al., 2017, Germany	To assess sleep quality in tracheotomized patients undergoing prolonged weaning	A cross-sectional study of 19 patients undergoing prolonged weaning at a specialized weaning unit of a pneumology department Groups: *“successful weaning”* and *“unsuccessful weaning”*	*Objective measurements:* PSG (10 pm – 6 am) Gas exchange monitoring *Subjective evaluation:* Sleep quality and SRI *Other parameters assessed:* Days on invasive MV	NO	7 patients (36.8%) *successful weaning* vs. 12 (63.2%) *unsuccessful weaning* Days on MV: 39 (SD 22) *successful weaning* vs. 187 (SD 335) *unsuccessful weaning*; p = 0.473	No significant difference in sleep quality or any items of the questionnaire between the groups		No difference was identified in sleep quality or questionnaire items between the successful and *unsuccessful weaning* groups.

* The study by Huttmann et al. (2017) objectively evaluated sleep before weaning initiation; additionally, subjective evaluation was performed in the morning following the termination of nocturnal measurements. APACHE II: Acute Physiology and Chronic Health Evaluation II, CI: confidence interval, GCS: Glasgow Coma Scale, MV: mechanical ventilation, PSG: polysomnography, SD: standard deviation, SRI: Severe Respiratory Insufficiency, VSH: Verran and Snyder-Halpern Sleep Scale

## RESULTS

### Characteristics of the studies

Of the six studies analyzed, four investigated the effect of patients' sleep on their weaning from mechanical ventilation [22,23,24,25], one assessed the presence of delirium and associated circadian rhythm changes during weaning [26], and one analyzed the relationship between sleep quality and the risk of reintubation [27]. The studies comprised small patient samples (19 to 94) treated in various hospital ICUs, pulmonology centers, or specialized weaning units. The total number of all patients included in the review was 324. The studies may be considered recent as the oldest were published in 2015.

All six studies stated the time on ventilator, ranging from three to 187 days. There were differences in airway management. One study evaluated sleep in patients with a tracheostomy tube [23]; others focused on patients receiving endotracheal intubation [22,25], and two studies involved both approaches [24,26].

The studies varied in when and how sleep was assessed. All studies on subjective sleep quality assessed sleep sometime after weaning initiation [23,24]. Of the studies using objective sleep measurement techniques, two assessed sleep architecture before a planned weaning attempt [22,23], one enrolled patients after a first failed weaning attempt and monitored sleep before a subsequent weaning attempt [25], and another assessed sleep after extubation concerning the risk of re-intubation [27]. Dessap et al. [26] collected 6-sulfatoxymelatonin (6-SMT) in the first 24 hours from weaning initiation. The last study included subjective sleep assessment after completion of overnight measurements and objective sleep assessment before weaning initiation [23].

### Studies assessing objective sleep quality

Five of the studies used objective measurement. In four studies with polysomnography (PSG), the timing of PSG recording varied (as stated in the previous paragraph) [22,23,25,27]. Of these, three studies [22,25,27] analyzed PSG recordings not only according to the conventional American Academy of Sleep Medicine (AASM) guidance [28] but also with alternative scoring systems for mechanically ventilated patients that added two new items to the AASM classification: atypical sleep, characterized by the absence of K complexes and sleep spindles in stage N2 sleep, and pathological wakefulness, characterized by altered electroencephalographic (EEG) reactivity and increased slow-wave activity [29]. Abnormal sleep patterns were present in 19% to 44% of PSG recordings. All three studies aimed to determine whether these abnormal sleep patterns affected the weaning course or increased the risk of re-intubation. Thille et al. [25] found that in a group of 45 mechanically ventilated patients, atypical sleep was associated with prolonged weaning. In contrast, a study by Dres et al. [23] failed to confirm the relationship between pathological wakefulness or atypical sleep and successful weaning attempts. The authors elaborated on the concept and used two additional EEG parameters to predict weaning success. The first was the odds ratio product (ORP), an index that evaluates sleep depth according to selected EEG parameters, ranging from 0 (very deep sleep) to 2.5 (full wakefulness). The second derived parameter was the intraclass correlation coefficient between the ORP in the right and left brain hemispheres, which compares sleep depth between the hemispheres. The likelihood of successful weaning was found to be highly correlated with the fraction of monitoring time spent in full wakefulness (ORP > 2.2). The second finding was that a poor correlation between sleep depth in the right and left hemispheres significantly predicted weaning failure. Finally, in their study of 52 patients, Thille et al. [27] explored whether atypical sleep impacted the risk of post-extubation respiratory failure or reintubation. The results showed no association with a higher risk of reintubation.

The negative impact of reduced or absent REM sleep on weaning from mechanical ventilation was documented in all studies examining REM sleep duration before extubation. Thille et al. [25,27] repeatedly described that the absence of REM sleep was associated with multiple reintubations and prolonged weaning. The authors of the other two studies, Dres et al. [22] and Huttmann et al. [23] found an association between reduced REM sleep and unsuccessful weaning.

Only one study measured sleep quality in non-sedated patients experiencing difficult weaning. Of the 19 evaluated patients, weaning was successful in 7 and unsuccessful in 12 [23]. In the other studies analyzing sleep, patients were sedated. Thille et al. [25] analyzed 45 patients on mechanical ventilation. Twenty-seven were in short weaning, and 18 were in prolonged weaning. Another study [27] assessed required for reintubation in 52 patients. Eight patients from this group had to be reintubated, and 44 were successfully extubated. The contribution of sedation to sleep changes (abnormal sleep patterns, absent or reduced REM sleep) was assessed in both studies [25,27] patients with these sleep changes were found to receive greater doses of sedation. Thus, it cannot be ruled out that sedation may also contribute to difficult weaning by impairing sleep before extubation.

Dessap et al. [26] explored the impact of the circadian rhythm on the development of delirium by measuring urinary levels of the melatonin metabolite 6-SMT during weaning (n = 70). Patients with delirium showed significantly reduced urinary excretion of the 6-SMT compared to patients without delirium.

### Studies assessing subjective sleep quality

In two studies, subjective sleep quality was subjectively assessed after liberation from mechanical ventilation, with patients retrospectively rating their sleep beforehand [23,24].

Chen et al.[24] used the validated *Verran and Snyder-Halpern Sleep Scale* [30]. In a group successfully weaned from ventilation within 72 hours, sleep quality was significantly better on all questionnaire items, and good sleep quality was also associated with successful weaning.

Huttmann et al. [23] assessed the quality and quantity of sleep in patients simultaneously with polysomnography (PSG) and their sleep questionnaire - containing four questions and found no difference in any items between weaning success and failure groups.

### Other factors affecting weaning assessed in the studies reviewed

Four studies included the impact of delirium on weaning, with the assessment tool being either the *Confusion Assessment Method For the Intensive Care Unit* [22,26] or the *Intensive Care Delirium Screening Checklist* with a cut-off value of ≥ 4 [25,27]. While three studies found delirium to be associated with more difficult weaning or reintubation [22,26,27], Thille et al. [25] reported no significant difference in weaning duration between patients with and without delirium. In both of their studies [25,27] failed to confirm a relationship between the presence of delirium and atypical sleep. One study evaluated the effect of urinary excretion of 6-SMT in patients undergoing weaning [26]. Urinary 6-SMT levels were lower in patients with delirium, suggesting an association between a disrupted circadian rhythm and the presence of delirium.

Muscle weakness, a potential impediment to weaning from mechanical ventilation, was studied [25,27]. Their subanalysis found muscle weakness to be an independent factor associated with prolonged weaning and significantly more frequent in patients with atypical sleep. However, the authors do not comment on the association between atypical sleep and ICU-acquired weakness.

Disease severity was described using the scoring systems *Acute Physiology and Chronic Health Evaluation II* (APACHE II) or *Sequential Organ Failure Assessment*. Most reviewed studies found an association between disease severity and weaning failure [26,22,24,25,27]. When looking for associations between disease severity and sleep, two studies failed to confirm a relationship between the presence of atypical sleep and the severity of the underlying disease [25,27]. According to Chen et al. [24], the relationship between sleep quality and disease severity is one of the factors affecting successful weaning of patients from mechanical ventilation.

## DISCUSSION

Mechanically ventilated patients have disturbed sleep for many reasons, including discomfort, multiple diagnostic, therapeutic, and nursing interventions, possible patient-ventilator asynchrony, the ICU environment, possible pain, forced positioning, and, of course, their illness itself [31]. This has been confirmed by studies using PSG to evaluate sleep in ventilated patients [32,33,34].

Evaluating the effect of sleep on weaning success is not straightforward. The findings from this review suggest that the association between good sleep and successful weaning is not strong, and its search is methodologically complicated due to the heterogeneity of cases. Some studies found that abnormal sleep patterns (atypical sleep, reduced REM sleep duration) were associated with weaning failure. However, many other factors were also present, limiting the interpretation of the associations found. Several studies have attempted to elucidate the role and cause of abnormal sleep patterns in patients on mechanical ventilation and to assess their occurrence as a predictor of their condition. An analysis of PSG recordings in 52 critically ill mechanically ventilated patients showed that atypical sleep patterns were rather significantly associated with higher mortality [35].

Sleep quality and weaning success in mechanically ventilated patients largely depend on sedation. To what extent does sedation cause changes in sleep architecture, and how sedation is related to the development of sleep apnea in extubated patients remains controversial. Literature findings are consistent in that patients receiving a combination of fentanyl and propofol showed a more significant proportion of abnormal PSG findings than non-sedated patients [36]. The effect of sedation on sleep architecture has also been confirmed by other studies [37,38].

Currently, there is a growing number of studies that not only look at the method and type of sedation itself but also compare different approaches (protocolized sedation, daily sedation interruption, periodic wake, and wean) that can significantly affect the duration of mechanical ventilation [39]. Although the use of opioids during mechanical ventilation improves the patient's tolerance to mechanical ventilation, the administration of higher doses increases the risk of post-extubation sleep apnea, potentially leading to respiratory failure [11,40]. Sleep apnea, both obstructive and central, is a risk factor for patients staying in the ICU. To what extent sleep apnea may influence weaning from mechanical ventilation remains unknown.

A growing body of evidence suggests impairment of circadian rhythm changes, both due to the severity of their illness and the ICU environment and practice (24-hour noise, light, interventions, etc.). Melatonin seems to be a good and easy-to-measure marker for circadian rhythm assessment in mechanically ventilated patients. It can be obtained directly from blood or saliva, which requires waking the patient. Another option is to measure urinary levels of the melatonin metabolite 6-SMT. The excretion of melatonin and the effect of its levels on weaning has not been investigated. There is a lack of more robust studies determining whether melatonin or any of its metabolites, could be evaluated as a marker predicting weaning procedure. Some authors have confirmed changes in secretion and pronounced temporal disorganization of patient profiles (a phase-delayed diurnal curve) [41,42]. Whether these diurnal variations may influence weaning success may be a topic for further research.

To maximally adapt to the physiology of breathing and minimize the effects of mechanical ventilation, new ventilation modes have been introduced in recent years that adapt to the patient's respiratory effort while preserving its variability, such as proportional assist ventilation and neurally adjusted ventilatory assist. They aim to achieve maximum patient-ventilator synchrony. Several authors have shown that these ventilation modes can positively influence sleep [43,44].

Sleep in mechanically ventilated patients highly depends on their overall condition, the most important factor influencing weaning success. This statement is consistent with the findings of a meta-analysis that included 65 observational studies evaluating various weaning predictors [45]. Significant physiological predictors of weaning include the number of breaths and the rapid shallow breathing index (i.e., the ratio of respiratory rate to tidal volume). At the same time, the meta-analysis reported that the APACHE II score, as obtained at hospital admission, appears to be a very promising tool for predicting weaning success.

Two studies in the present review showed that muscle weakness in critically ill patients was an important factor influencing weaning success (extubation). According to a 2015 study by Hermans and Van den Berghe, the incidence of muscle weakness ranges from 26% to 67%, depending on the duration of mechanical ventilation, and its presence is associated with difficult weaning [46]. Diaphragmatic dysfunction has been observed in muscle weakness, and ultrasound is a promising tool for predicting unsuccessful weaning [47].

Delirium is associated with difficulty weaning or extubation [48,49]. In most reviewed studies, delirium was directly related to problems during weaning, such as ventilator-associated pneumonia, more sedation administered, or the need for catecholamine administration.

### Limitations of the study

This review has several limitations: (1) different groups of patients with different severity of the underlying disease, (2) lack of differentiation of ventilation modes, (3) different methods of airway management (endotracheal tube, tracheostomy), (4) limited assessment of the effect of sedation, (5) failure to include other factors associated with the ICU environment (noise, light, nursing interventions), and (6) a small number of studies addressing the topic.

## Conclusion

Patients with difficult weaning had a higher prevalence of abnormal sleep patterns, shortened REM sleep duration, and reduced melatonin excretion. Sleep highly depends on the overall disease severity, contributing most to successful weaning. Despite the limited impact of sleep quality on weaning, the authors emphasize that sleep management in mechanically ventilated patients is part of a comprehensive approach in intensive care.

## Implications for practice

Patients with difficult weaning had a higher prevalence of abnormal sleep patterns, shortened REM sleep duration, and reduced melatonin excretion.

Sleep abnormalities are an understudied aspect of difficult weaning of patients from mechanical ventilation.

Muscle weakness is an independent factor associated with prolonged weaning and is significantly more frequent in patients with atypical sleep.
